# Use of Organic Acids in Bamboo Fiber-Reinforced Polypropylene Composites: Mechanical Properties and Interfacial Morphology

**DOI:** 10.3390/polym13122007

**Published:** 2021-06-19

**Authors:** Lety del Pilar Fajardo Cabrera de Lima, Cristian David Chamorro Rodríguez, José Herminsul Mina Hernandez

**Affiliations:** 1 Grupo Tribología, Polímeros, Metalurgia de Polvos y Transformaciones de Residuos Sólidos, Universidad del Valle, Calle 13 No. 100-00, 76001 Cali, Colombia; 2 Grupo Desarrollo, Innovación e Investigación en Diseño, Universidad del Valle, Calle 13 No. 100-00, 76001 Cali, Colombia; cristian.chamorro@correounivalle.edu.co; 3 Grupo Materiales Compuestos, Universidad del Valle, Calle 13 No 100-00, 76001 Cali, Colombia; jose.mina@correounivalle.edu.co

**Keywords:** bamboo fibers, composites, organic acids, coupling agent, mechanical properties

## Abstract

In obtaining wood polymer composites (WPCs), a weak interfacial bonding can cause problems during the processing and affect the mechanical properties of the resulting composites. A coupling agent (CA) is commonly used to solving this limitation. To improve the interfacial bonding between bamboo fiber (BF) and a polypropylene matrix, the effect of three organic acids on the mechanical properties and interfacial morphology were investigated. The BF/PP composites were prepared in five families: the first without CA, the second using a maleic anhydride-grafted polypropylene coupling agent, and the third, fourth, and fifth families with the addition of organic acids (OA) tricarboxylic acid (TRIA), hexadecanoic acid (HEXA), and dodecanoic acid (DODA), respectively. The use of OA in BF/PP improved the interfacial adhesion with the PP matrix, and it results in better mechanical performance than composites without CA. Composites coupled with MAPP, TRIA, DODA, and HEXA showed an increase in Young’s modulus of about 26%, 23%, 15%, and 16% respectively compared to the composite without CA incorporation. In tensile strength, the increase in composites with CA was about 190%, while in the flexural modulus, the coupled composites showed higher values, and the increase was more in composites with TRIA: about 46%. The improvement caused by tricarboxylic acid was similar to that promoted by the addition of maleic anhydride-grafted polypropylene (MAPP).

## 1. Introduction

The growing concern for the environment has led to the search for sustainable materials that are capable of replacing conventional materials in certain applications. This search has generated a great interest in the use of natural, recyclable, renewable, and biodegradable materials. The interest in natural fiber-reinforced composites (NFRC) has resumed, with the purpose of improving the thermal and mechanical properties and reducing the costs in the composition of polymeric materials, in addition to diminishing the generation of effluents and/or polluting wastes [1].

In recent decades, there has been a significant increase in the development of composites reinforced with lignocellulosic natural fibers, as these have several advantages, including having good strength, low specific gravity and biodegradability, while they are also natural and affordable [2,3].

Agroindustrial residues (coconut, rice husk, sugarcane bagasse, and itauba, among others) [4] and fibers such as flax, jute, hemp, and sisal are among the most used natural fibers in thermoplastic composites [5]. Other lignocellulosic fibers such as bamboo have also been explored as a composite reinforcement. This genus constitutes one of the most extensive botanical families, with many uses and applications. Bamboo is well known for its quick growth (which is ready for use within 3–4 years) and ability to be renewed without replanting [6]. Composed mainly of 73.83% cellulose, 10.15% lignin, 12.49% hemicellulose, 0.3% pectin, and 3.16% aqueous extract, other constituents are protein, fat, and tannins [1]. In this paper, composites of polypropylene (PP) matrix were reinforced with the bamboo specie *Guadua angustifolia* fibers; this specie is known for its excellent characteristics for civil construction, but there are few studies of this specie as reinforcement in thermoplastic composites. 

In obtaining NFRCs, one drawback is the polarity difference between materials, i.e., the polar and hydrophilic nature of the lignin-cellulosic fibers and the nonpolar characteristic of most thermoplastics. This polarity difference leads to a weak interfacial adhesion [1,2,3,4,5,7].

The interface between a lignocellulosic material and a thermoplastic matrix has a great influence on the mechanical properties, since the load transfer from the matrix to the fiber occurs through this interface. To improve the matrix–reinforcement interaction, coupling agents that promote greater adhesion between the phases can be used. Polymers such as PP have been modified with maleic anhydride [8,9,10]. The use of MAPP is a common method used to treat the incompatibility between the polymeric matrix and plant fiber-reinforced composites and chemically alter the resin, adding polar groups to the chain by means of the grafting reaction with acrylic acid or maleic anhydride [8]. Maleic anhydride has a polymer structure that easily links to other functional groups; it has carboxyl and hydroxyl groups that allow it to bond with other functional groups when it is grafted into the polymer structure [8]. The effect on the incorporation of the reinforcement to the maleated polypropylene matrix can be understood by the esterification reaction, where hydrogen bonds of the cellulose interact with the MAPP to reduce the material’s susceptibility to humidity. In this way, the hydroxyl groups of the fiber will be chemically bound to the MAPP groups, creating a less hydrophilic reinforcement [10]. It should be mentioned that MAPP is a CA of synthetic origin; despite being widely used in obtaining NFRCs, it is expensive, difficult to manufacture, and not biodegradable [11]. As mentioned above, the concern for the depletion of fossil fuels and the interest with the preservation of the environment is of universal interest; in this sense, the use of materials from organic sources is increasingly important. The use of natural sources components may be identified as emerging trends in composite materials [12], since they allow contributing to obtain ecological materials; additionally, they present economic and social advantages. Some authors have reported that the use of green coupling agents in vegetable fibers-based thermoplastic composites can be comparable with the MAPP and silane coupling agent [13,14]; natural oils have been investigated as coupling agents to improve the adhesion at the fiber/matrix interface and have shown to promote an increase in the thermal stability of the composite formed, concluding that these organic oils have an effect similar to that of MAPP [15,16,17]; some authors have reported that the use of palmitic acid as a coupling agent in place of silane is a renewable green chemical [18]. Organic acids are called carboxylic acids and are found in animal and plant constituents, or they can also be obtained by chemical synthesis. They are usually used in the chemical, pharmaceutical, food, and animal nutrition industries. They are natural organic compounds of medium and long chains and have a chemical structure similar to that of synthetic coupling agents, with the advantage that they are of organic origin and do not pollute the environment [10]. In addition to coming from renewable sources, they are abundant in nature and much cheaper compared to the commercial coupling agents that are commonly used. The main novelty of this study is the use of coupling agents of natural source with similar effect to those presented by the MAPP; they are a more economical option and have environmental advantages compared to use of coupling agents of petrochemical source. The organic acids used in this study are as follows: tricarboxylic acid is a fatty acid produced by the fermentation process, using sugar as a substrate; dodecanoic acid is a 12-carbon medium-chain saturated fatty acid linked to a molecule of hydrogen; the main sources of this acid are palm and coconut oils, and the hexadecanoic acid in a saturated fatty acid does not have double bonds between the carbons; it is a medium-chain 16-carbon structure that is obtained mainly from palm oil.

To compare the effects of these acids in the BF/PP composite, their mechanical properties, such as tensile strength, modulus of elasticity, flexural strength, flexural modulus, and impact strength were evaluated to determine their mechanical performance. 

The main objective of this study was to evaluate the use of these organic acids as coupling agents in BF/PP composites to propose an alternative to the use of synthetic coupling agents.

## 2. Materials and Methods

### 2.1. Materials

**Figure 1:**
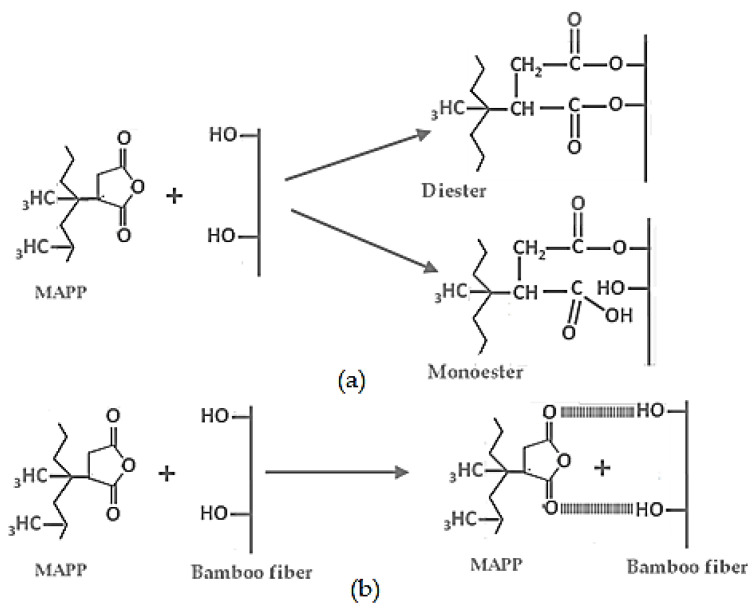

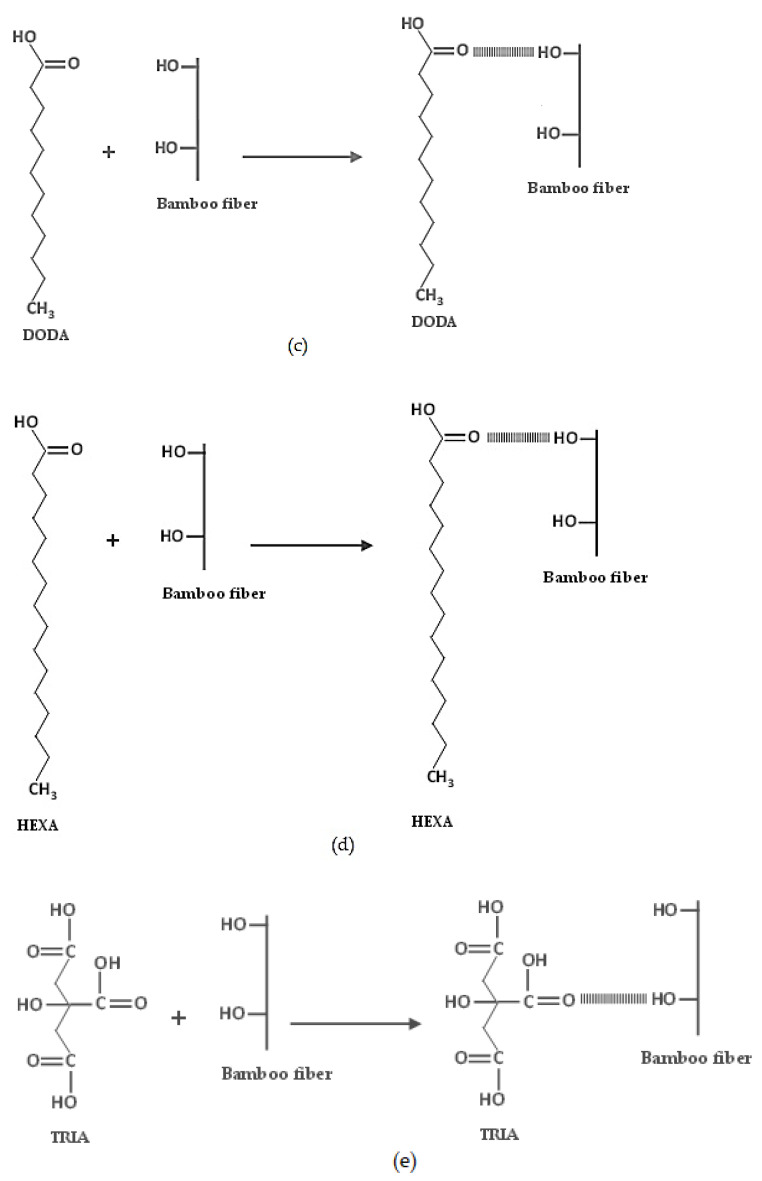
Schematic representation of (**a**) reaction between MAPP and bamboo hydroxyl groups, (**b**) interaction between MAPP and bamboo hydroxyl groups, (**c**) interaction between DODA and bamboo hydroxyl groups, (**d**) interaction between HEXA and bamboo hydroxyl groups, and (**e**) interaction between TRIA and bamboo hydroxyl groups.

The materials used in this study were as follows: PP (MFI = 45 g/10 min) manufactured and donated by Braskem (Porto Alegre, RS, Brazil); MAPP manufactured by DuPont Fusabond MZ-109D (Barueri, SP, Brazil); tricarboxylic acid (TRIA) and dodecanoic acid (DODA) manufactured by Neon Comercial Ltd. (Suzano, SP, Brazil); hexadecanoic acid (HEXA) manufactured by Dinâmica-Química Contemporânea Ltd. (Indaiatuba, SP, Brazil); bamboo fiber (BF) of the *Guadua angustifolia* species with particle sizes of 250 μm (bamboo flour), from (Popayán)-Colombia derived from manufacturing product residue. All reagents used were analytical grade. Figure 1 shows examples of the possible interaction between MAPP, DODA, HEXA, and TRIA with the hydroxyl groups of cellulose present in bamboo fibers.

### 2.2. Processing Conditions

The materials were weighed separately using an AY-220 analytical balance and prepared with the compositions described in Table 1.

The materials were manually mixed according to the established mass relationships before processing. An internal mixer chamber HAAKE Rheomix OS Polylab (Thermo Fisher Scientific, Karlsruhe, Germany) was used to obtain the composites. The materials were processed for 5 min at a temperature of 180 °C, with a rotational speed of 60 rpm. The specimens for characterization were injection-molded, using a mini-injector HAAKE Minijet II, (Thermo Fisher Scientific, Karlsruhe, Germany), at a temperature of 195 °C, a mold temperature of 40 °C, and a pressure of 400 bar.

## 3. Characterization

### 3.1. Mechanical Tests

The determination of the tensile and flexure mechanical properties of the composites BF/PP, BF/PP/MAPP, BF/PP/TRIA, BF/PP/DODA, and BF/PP/HEXA were carried out on a Instron model 4200 universal testing machine (Instron, São Jose dos Pinhais, PR, Brazil); tensile tests were performed according to ASTM D638 [19] using a 5 kN cell load and a speed of 2 mm/min with type IV specimens, and flexure tests were carried out according to ASTM [20], D790 using a 5 kN cell load and a speed of 1.35 mm/min. Impact tests were carried out on a CEAST model Impactor II with a 2.75 J hammer, without an entangler, according to ASTM D256. Seven specimens were used for each formulation of the mechanical tests, and the average of the data was taken for the analysis.

### 3.2. Morphological Test

The morphological characterization in the fiber–matrix interface of the composite was carried out in a Jeol Brand Scanning Electron Microscope (SEM) model JSM 6060 (Jeol, São Paulo, SP, Brazil) with 400× magnification and under an accelerating voltage of 15 kV. The fracture surfaces of the composites were evaluated after the tensile test, and the samples were metallized with gold 24 h before performing the analysis.

## 4. Results and Discussion

### 4.1. Mechanical Properties

**Figure 2:**
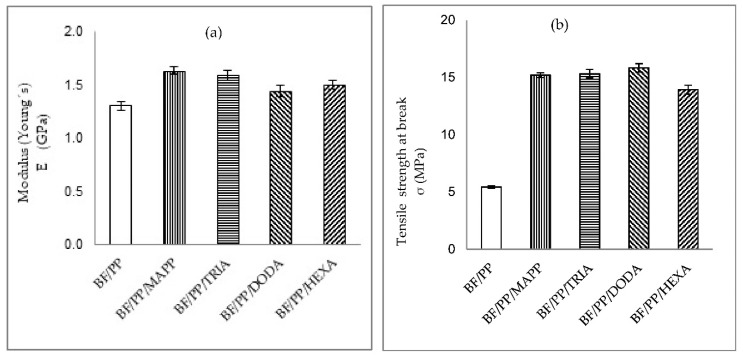
Results of the tensile test of the composites without and with CA: (**a**) Young’s modulus and (**b**) tensile strength at break.

**Figure 3:**
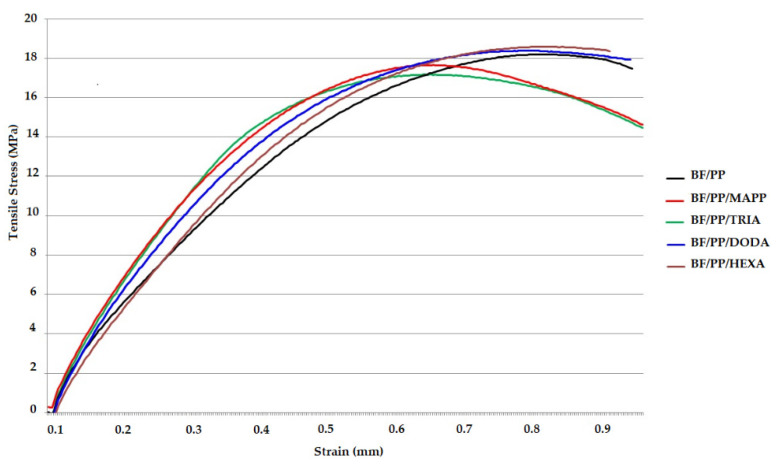
Tensile stress–strain curves of the composites without and with CA.

Figure 2a shows the results of Young’s modulus, where the composites with CA were found to have higher values than those without CA, indicating an increase in the stiffness. The incorporation of coupling agent increases the modulus of elasticity when compared to the non-CA composite, as confirmed by the stress–strain curves in Figure 3. 

The composites coupled with MAPP, TRIA, DODA, and HEXA showed higher values, 26%, 23%, 15%, and 16% respectively compared with BF/PP. Figure 2b shows the results of the tensile strength at break, where a notable difference is observed between the compatibilized composites and those without CA. 

The composites with CA presented an approximate increase of 192% (with statistically equal values); these results were analyzed with the statistical tool ANOVA of single factor 95% reliability level, in the Software Statistical. The coupling agents interfere by increasing the strength at the break [7,8,9,16,21], indicating a greater interaction at the fiber–matrix interface [22,23]. This increase is probably due to the secondary bonds generated between the carbonyl group of the CA and the hydroxyl groups on the surface of the fibers [17,21]. 

The best performance presented by the composites with CA (MAPP and organic acids) may be due to the increase in interfacial interactions between the polymer and the fiber; the coupling agent has a chemical structure that improves the adhesion at the polymer/fiber interface. Tensile strength is influenced by an increase in interfacial adhesion; in this study, the coupled composites showed a similar trend for tensile modulus and tensile strength; other authors also reported this trend [13,14]. In similar studies, some authors reported that a good dispersion of the fibers in the matrix improves the mechanical performance of the composites [8,24]. Other authors observed that the composites coupled with MAPP presented a better strength transfer through the matrix for the reinforcement, which is probably because of the mechanical fixation or chemical interactions between the anhydride and hydroxyl groups of the cellulose in the BF/PP interface [25,26].

Among the composites with CA, those coupled with MAPP and TRIA showed slightly higher values. The presence of maleic anhydride in the MAPP increased the number of bonds interacting with the hydroxyl groups of the bamboo fiber through the formation of diesters and monoesters, which provide a better interfacial zone [22]; interdiffusion is the main mechanism of adhesion between the polypropylene chains belonging to the MAPP-modified fibers and the macromolecules that are part of the polypropylene that makes up the matrix of the composite. TRIA showed similar behavior to MAPP, which may be related to the chemical structure of the OA, particularly the polar group, as the degree of compatibility of the matrix with the reinforcement is related to the chemical structure and nature of the CA [27,28]. The secondary interactions of the TRIA with the bamboo fibers, mainly hydrogen bonds, promote the formation of a coating that provides a more hydrophobic character to the fibers and favors the possibilities of a more significant wetting with the polypropylene matrix, thus generating an adhesion mechanism by mechanical anchoring in the interfacial zone and taking advantage of the intrinsic surface roughness of the bamboo fibers. Additionally, an internal lubrication phenomenon occurs in the fibers, which results in a better distribution of the fibers in the matrix, as explained above. Tricarboxylic acid has a fairly polar characteristic, having a greater affinity with fiber, which is very different from DODA and HEXA, where both have long chains with CH_2_ groups and only one hydroxyl group (OH), which means that their polarity is weak compared to TRIA and MAPP. 

**Figure 4:**
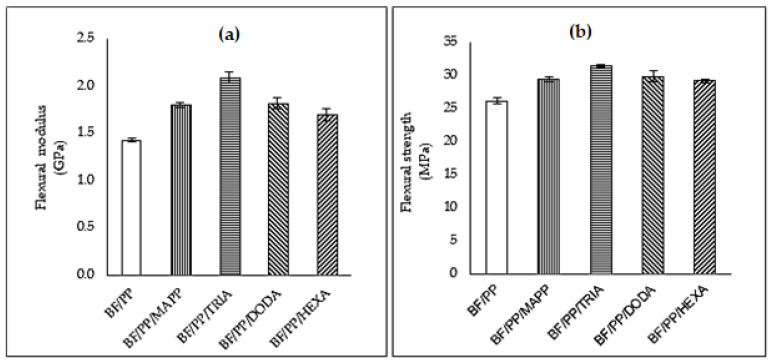
Results of the flexural test of the composites without CA and with CA: (**a**) modulus and (**b**) flexural strength.

The results of the flexural test are shown in Figure 4; here, it can be seen that in all composites where the CA was incorporated, significant increases in modulus (Figure 4a) and flexural strength (Figure 4b) were achieved. 

Among the materials characterized, the BF/PP/TRIA composites stand out, where flexural modulus was obtained with increases of 16%, 15%, and 24% compared to the results of the BF/PP/MAPP, BF/PP/DODA, and BF/PP/HEXA composites, respectively.

The increase in the flexural strength (Figure 4b) values are related to the reduction of the mobility of the polymer chains caused by the increase in interfacial adhesion between the polymer/fiber matrix [29] due to generating mechanical interlocking and/or interdiffusion mechanisms and a better distribution of the bamboo fibers in the composite matrix. The presence of TRIA may have generated a greater adhesion between the reinforcement and polymeric matrix, which was probably due to the formation of hydrogen bonds between the bamboo fiber hydroxyl groups and the TRIA carbonyl or hydroxyl groups and interlinking between PP chains with this organic acid. Similar behavior was observed by Poletto et al. [26], where octanoic acid was used as the coupling agent in polypropylene/wood fiber composites. This finding may indicate that the presence of TRIA allowed a greater amount of force to be transferred from the polymer to the fiber, which increases the flexural strength.

**Figure 5:**
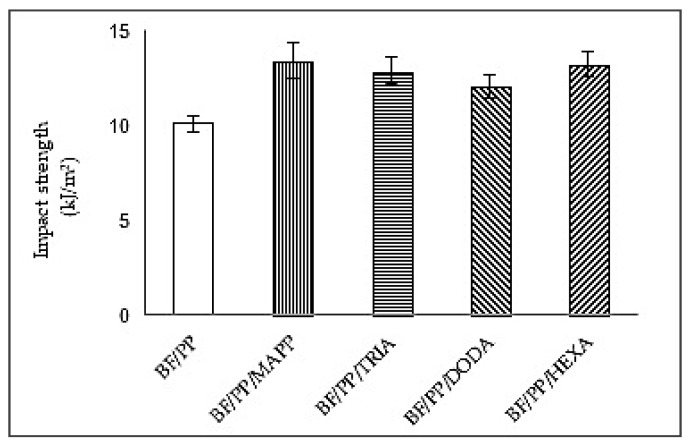
Results of the impact strength test of the composites without CA and with CA.

Figure 5 shows the results of the impact strength of the composites without and with CA. The use of CA promoted impact strength; other authors also reported these observations [6,30]. The BF/PP/MAPP composite was the one with the highest value, followed by the BF/PP/HEXA BF/PP/TRIA and BF/PP/DODA (these composites with statistically similar values). The interfacial adhesion between the bamboo fiber and the polypropylene matrix was favored by incorporating the different coupling agents studied. This behavior was reflected in the increase of the material’s strength and modulus compared to the composite without CA incorporation. These explanations are consistent with the results showed above in tensile stress–strain curves of the composites without and with CA (Figure 3).

Additionally, the increase of the impact resistance that was evidenced for the composite with CA was due, probably, to the establishment of mechanisms of interfacial adhesion promoted by the generation of new physical interactions (in their majority hydrogen bonds) that improved the union fiber–matrix but without restricting the possibility of energy absorption when the material was put under a dynamic load during the realized impact test.

It is important to highlight that the amount of the different coupling agents used (MAPP, DODA, TRIA, and HEXA) was not very high (3%), so the interfacial zone was not very stiff, achieving a higher toughness in the modified material compared to the non-CA composite. Similar behavior was obtained by Zhou et al. (2013) [29], who also reported increases in impact resistance in a PP composite reinforced with bamboo fibers for MAPP ratios of up to 9%; from this point on, with the further additions, they found that this mechanical property was reduced.

Composites with CA showed greater resistance to impact. This may indicate that the presence of MAPP and organic acids promoted a greater adhesion between the fiber and the polymer; this is also confirmed by the mechanical properties mentioned above. The difference of the values in the composites coupled with organic acids may be related to their chemical structures as mentioned in the behavior of the tensile test. The improvement in mechanical strength can be attributed to the hydrophilic anhydride groups of the CA that are prone to compatibility with bamboo fibers, thus promoting the dispersion of the fiber in the matrix [5]. 

The results of the mechanical properties of the composites evaluated are shown in Table 2. In the most of the mechanical properties evaluated, no statistical differences were observed between composites with CA, excepting the results of flexural modulus, where the use of TRIA promoted the highest value, according with the calculation of the statistical tool ANOVA of single factor.

### 4.2. Morphology

**Figure 6:**
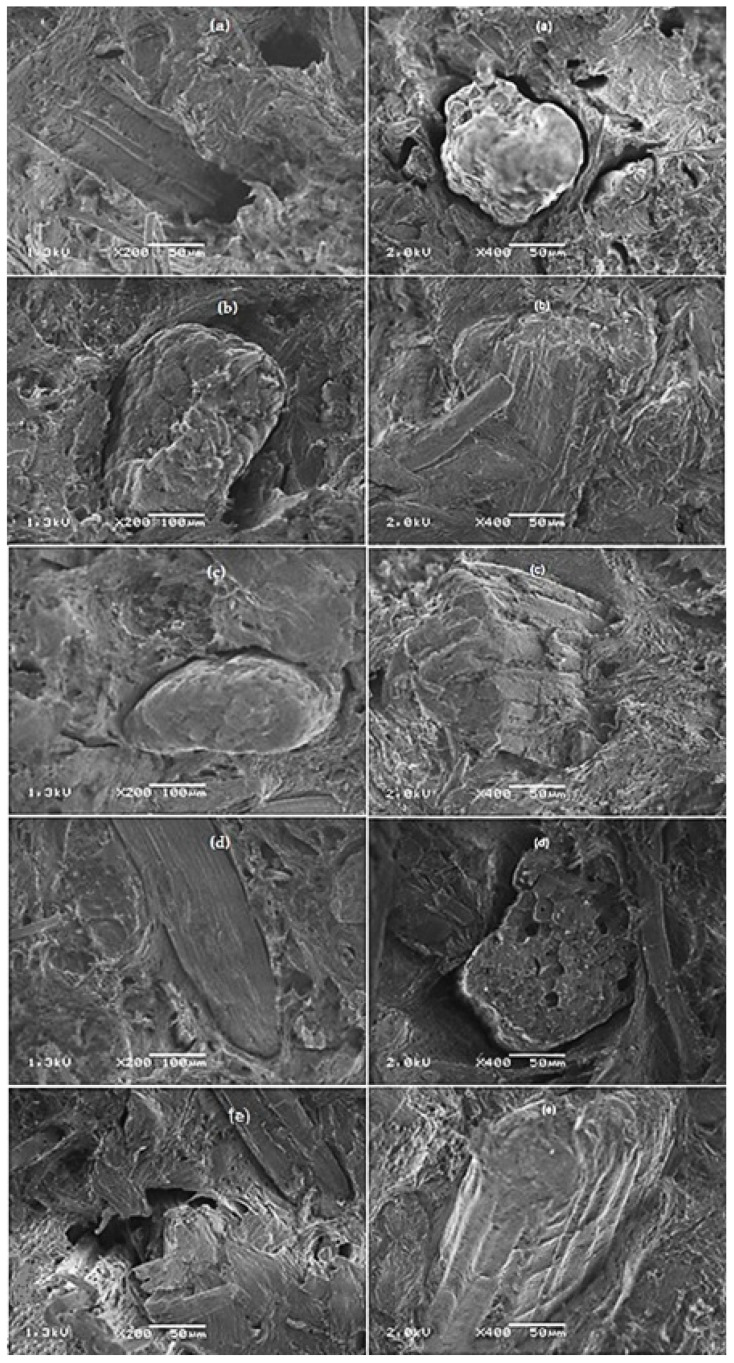
SEM micrographs of the fracture surface after the tensile test: (**a**) BF/PP ×200 and ×400; (**b**) BF/PP/MAPP ×200 and ×400; (**c**) BF/PP/TRIA ×200 and ×400; (**d**) BF/PP/DODA ×200 and ×400; (**e**) BF/PP/HEXA ×200 and ×400.

Figure 6 shows SEM micrographs of the fracture surface after the tensile test of the evaluated composites. In Figure 6a, the composite without CA is shown; the gap left by the fiber dislocation at the time of fracture can be observed, which clearly shows that the fiber is not wet by PP, which is indicative of a weak interfacial fiber/matrix adhesion [21,29,30]. In Figure 6b–e, the composites with CA show that the fiber is wet by PP; in Figure 6b,c, the surfaces of the BF/PP/MAPP and BF/PP/TRIA composites, respectively, present greater adherence; this finding confirms that these composites have greater tensile strengths, which indicates that the presence of CA improved the interaction of the bamboo fiber with the PP matrix. Other authors also observed that the presence of CA promotes the fiber/matrix interaction [29,30,31,32,33,34], and similar observations have been mentioned with the use of OA as a coupling agent [15,16,17,18,26].

In general, these observations are consistent with the results of the mechanical properties, where the BF/PP/MAPP and BF/PP/TRIA composites showed surfaces with strong interactions and increased interfacial adhesion. Chattopadhyay et al. [34] mentioned that the anhydride group of MAPP covalently links with the hydroxyl groups of the bamboo fibers forming an ester linkage, while the nonpolar part of MAPP becomes compatible with the matrix (PP), lowers the surface energies of the fibers, and hence, increases their wettability and dispersion within the matrix. Other authors observed that thermoplastic composites reinforced with vegetable fibers and coupled with MAPP showed strong interactions and fixing points between the polymeric matrix and the plant fiber [21]. Catto [27], Liu [33], and Mi [6] also observed that composites coupled presented fractured surfaces with better fiber/matrix interaction when compared with uncoupled composites. Poleto et al. [16] observed that the presence of caprylic acid in recycled PP/wood fiber composites promoted a strong interfacial interaction.

## 5. Conclusions

In general, the results showed that the presence of the CA influenced the characteristics of the BF/PP composites in such a way that the CA-containing composites showed greater interaction between the polymer and fiber and, consequently, better mechanical properties. The composites coupled with MAPP and those coupled with TRIA showed a modulus of elasticity 25% higher than the composite without CA.

Among the composites compatibilized with organic acids, different levels of compatibilization were observed, which were due in large part to the chemical structure of these acids. Notably, tricarboxylic acid, which provided a better dispersion of the fibers in the polymer matrix, significantly promoted the BF/PP interfacial adhesion. The composites compatibilized with tricarboxylic acid had a higher flexural modulus when compared with commercial CA and other organic acids.

The use of tricarboxylic acid is presented as an environmentally sensitive alternative of organic origin compared to the synthetic commercial coupling agent MAPP.

Regarding the use of DODA and HEXA as coupling agents, it may be necessary to study different percentages of incorporation to obtain a more efficient compatibility.

## Figures and Tables

**Table 1:** Formulations of composites.

Formulation	BF (%)	PP (%)	Coupling Agent (3%)
BF/PP	30	70	Without/CA
BF/PP/MAPP	30	67	MAPP
BF/PP/TRIA	30	67	Tricarboxylic acid
BF/PP/DODA	30	67	Dodecanoic acid
BF/PP/HEXA	30	67	Hexadecanoic acid

**Table 2:** Summary results of the mechanical properties of BF/PP composites without and with CA.

Samples	Modulus (Young’s) E (GPa)	Tensile Strength at Break σ (MPa)	Flexural Modulus E (GPa)	Flexural Strength Break σ (MPa)	Impact Strength (kJ/m^2^)
BF/PP	1.30 ± 0.13 ^a^	5.43 ± 0.36 ^c^	1.43 ± 0.01 ^e^	26.1 ± 0.46 ^h^	10.10 ± 0.45 ^j^
BF/PP/MAPP	1.63 ± 0.10 ^b^	15.83 ± 0.43 ^d^	1.80 ± 0.02 ^f^	29.4 ± 0.31 ^i^	13.42 ± 0.95 ^k^
BF/PP/TRIA	1.59 ± 0.06 ^b^	15.32 ± 0.32 ^d^	2.09 ± 0.04 ^g^	31.5 ± 0.22 ^i^	12.89 ± 0.70 ^k^
BF/PP/DODA	1.49 ± 0.10 ^b^	15.19 ± 0.22 ^d^	1.82 ± 0.02 ^f^	29.9 ± 0.80 ^i^	12.08 ± 0.63 ^k^
BF/PP/HEXA	1.50 ± 0.05 ^b^	13.92 ± 0.67 ^d^	1.69 ± 0.01 ^f^	29.2 ± 0.29 ^i^	13.24 ± 0.67 ^k^

^a,b,c,d,e,f,g,h,i,j,k^ Equal letters in the same column indicate that there are no significant differences between samples.

## Data Availability

The data presented in this study are available on request from the corresponding author.
